# The Role of Lactic Acid Adsorption by Ion Exchange Chromatography

**DOI:** 10.1371/journal.pone.0013948

**Published:** 2010-11-11

**Authors:** Qiang Gao, Fabao Liu, Tongcun Zhang, Jian Zhang, Shiru Jia, Changyan Yu, Kunyu Jiang, Nianfa Gao

**Affiliations:** Key Laboratory of Industrial Microbiology, Ministry of Education & Tianjin City, College of Biotechnology, Tianjin University of Science and Technology, Tianjin, People's Republic of China; University of Houston, United States of America

## Abstract

**Background:**

The polyacrylic resin Amberlite IRA-67 is a promising adsorbent for lactic acid extraction from aqueous solution, but little systematic research has been devoted to the separation efficiency of lactic acid under different operating conditions.

**Methodology/Principal Findings:**

In this paper, we investigated the effects of temperature, resin dose and lactic acid loading concentration on the adsorption of lactic acid by Amberlite IRA-67 in batch kinetic experiments. The obtained kinetic data followed the pseudo-second order model well and both the equilibrium and ultimate adsorption slightly decreased with the increase of the temperature at 293–323K and 42.5 g/liter lactic acid loading concentration. The adsorption was a chemically heterogeneous process with a mean free energy value of 12.18 kJ/mol. According to the Boyd^_^plot, the lactic acid uptake process was primarily found to be an intraparticle diffusion at a lower concentration (<50 g/liter) but a film diffusion at a higher concentration (>70 g/liter). The values of effective diffusion coefficient *D_i_* increased with temperature. By using our Equation (21), the negative values of *ΔG°* and *ΔH°* revealed that the adsorption process was spontaneous and exothermic. Moreover, the negative value of *ΔS°* reflected the decrease of solid-liquid interface randomness at the solid-liquid interface when adsorbing lactic acid on IRA-67.

**Conclusions/Significance:**

With the weakly basic resin IRA-67, *in situ* product removal of lactic acid can be accomplished especially from an open and thermophilic fermentation system without sterilization.

## Introduction

Lactic acid is a very important organic acid with a wide range of applications in the food, pharmaceutical, leather and textile industries. It is classified as GRAS for general-purpose food additives by the FDA of USA. Because of the properties such as mild acidic taste, nonvolatility, lack of odor and bacteriostasis, lactic acid is used as an acidulant, taste enhancer, pH regulator and preservative in the food industry. Sodium lactate, calcium lactate, ethyl lactate and the biodegradable polymer, polylactic acid (PLA), have already been used in pharmaceutical and cosmetic applications, especially PLA for medical applications such as controlled-release drugs, surgical sutures, and prostheses [1]. Lactic acid can be conventionally produced by chemical or enzymatic synthesis, but is mainly by fermentation using lactic acid bacteria, such as some *Lactobacillus* and thermophilic *Bacillus* species, or the fungus *Rhizopus oryzae* [2], [3].

To date, many attempts have been made to improve the fermentation productivity which suffers from serious product inhibition. CaCO_3_ is one of the traditional additives for product precipitation and neutralization. It is added into lactic acid fermentation broths to neutralize the free lactic acid and thus to minimize product inhibition. Although in downstream processing, the raw calcium lactate is concentrated, crystallized, separated from dissolved impurities by filtration, and acidified with sulfuric acid, some impurities are still in the cake and quite a lot of calcium lactate is washed away and becomes a pollutant to our environment.

Recently, several novel approaches have been investigated for lactic acid recovery, such as solvent extraction, electrodialysis, etc. However, due to the hydrophilic nature, lactic acid is hardly extractable by the common organic solvents. Although reactive extraction has been considered to be an interesting alternative to the conventional process, it needs high amounts of solvents and the toxic effects of the extractants and diluents restrict its application. With the development of the membrane process, electrodialysis fermentation is promising since it can remove the lactic acid from the broth and maintain the pH in a proper value. However, the approach also encounters many problems such as membrane fouling, deionization of the fermentation broth and a higher operating cost [4].

Ion exchange technique has been widely used in bio-separations. As for lactic acid adsorbents, they must possess the important characteristics of high capacity and selectivity for lactic acid over water and substrate, regenerability, and biocompatibility with microorganisms. Recently, many different adsorbents have been investigated for lactic acid removal from fermentation broth such as poly(4-vinylpyridine) resin (PVP), Reillex 425, MWA-1, VI-15, Amberlite IRA-400, IRA-420, IRA-900, IRA-92, IRA-35 and IRA-96, etc [5]–[8]. A few papers [8], [9], [10] have reported that IRA-67 resin is a useful adsorbent for lactic acid or some other organic acid extraction. However, little systematic study has been done on the mechanism and adsorption efficiency of lactic acid under different operating conditions.

The purpose of this work is to investigate the adsorption kinetics of Amberlite IRA-67 by examining temperature, resin dose and lactic acid concentration. Also, we examine the adsorption mechanisms at different temperatures and lactic acid loading concentrations. These data could be very useful for the *in situ* removal of lactic acid especially by thermophilic *Bacillus* producers in unsterilized media. Adsorption thermodynamics is also studied in this condition.

## Results

### Adsorption kinetics

**Figure 1:**
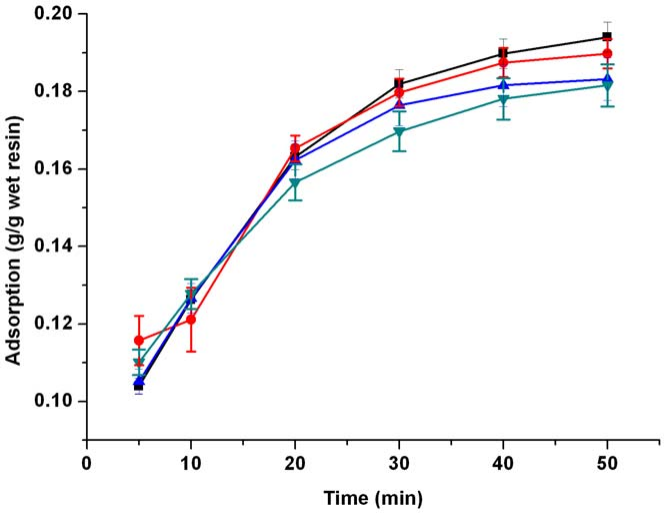
Effects of contact time and temperature on adsorption of lactic acid by IRA-67. Symbols: ▪, 293K; •, 303K; ▴, 313K; ▾, 323K.

Information on the kinetics of lactic acid uptake by IRA-67 resin was needed for selecting optimum operating conditions for *in situ* removal of lactic acid from the aqueous environment. Figure 1 showed the changes in adsorption of lactic acid at different contact times at 293, 303, 313 and 323K, respectively. As seen from the chart, the value of *q_t_* (g lactic acid/g wet resin) decreased somewhat with the increase of operating temperature, but the effect was not significant. Considering the possible bacterial contamination during the process of *in situ* removal of the product lactic acid, 323K was thought to be a promising operating temperature which would sharply decrease such contamination. As to the reversible reaction of 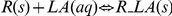, the change of lactic acid concentration (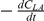) is relative with (*C_LA_−C_e_*)^n^ and n is 1 or 2 [11].

When n  =  1, then:
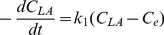
(1)After calculation, the following equation is obtained:
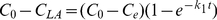
(2)Substituting Equation (2) into Equation (26), then:
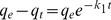
(3)where *R(s)* stands for raw resin (*R*) in solid state (s), *LA* (*aq*) represents the free lactic acid (*LA*) in liquid state (*aq*), *R_LA* stands for resin occupied by lactic acid, *C_0_* is the initial concentration of lactic acid (g/liter), *C_LA_* and *C_e_* are the concentrations of lactic acid at time *t* (min) and equilibrium (g/liter) respectively, *q_e_* is the amount of lactic acid adsorbed at equilibrium (g/g wet resin), *q_t_* is the amount of lactic acid adsorbed at time *t* (min), and *k_1_* is the equilibrium rate constant of the pseudo-first order adsorption.

The rate constants were obtained via the straight line plots of ln(*q_e_−q_t_*) vs *t* under different experimental conditions. The values of *k_1_* and correlation coefficient 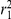 were calculated from these plots (Table 1).

**Table 1:** Kinetic parameters of pseudo-first order and pseudo-second order reaction at various temperatures.

TemperatureK	*q_e,exp_* g/g_wr_	Pseudo-first order			Pseudo-second order		
		*k_1_*	*q_e1,cal_* g/g*_wr_*	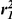	*k_2_*	*q_e2,cal_* g/g_wr_	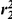
293	0.2093	0.0399	0.1145	0.940	0.513	0.2144	0.998
303	0.2014	0.0472	0.0991	0.900	0.575	0.2109	0.994
313	0.1909	0.0565	0.1000	0.954	0.581	0.2040	0.999
323	0.1820	0.107	0.1766	0.923	0.624	0.1990	0.999

g_wr_ in Tables 1–3 stands for “g wet resin”.

When n  =  2, then:
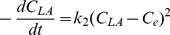
(4)After calculation, the following Equation (5) is derived:
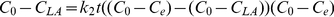
(5)Substituting Equation (5) into Equation (26), then:
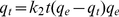
(6)Equation (6) can be rearranged as a linear form:
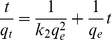
(7)and
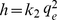
(8)where *k_2_* is the rate constant of the pseudo-second order equation, and *h* is the initial sorption rate (g/g wet resin per min) [12].

**Figure 2:**
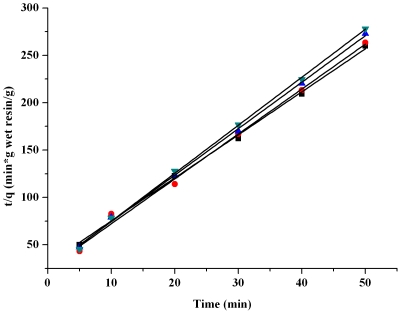
Linear plots of *t/q_t_* vs *t* for the pseudo-second order model. Symbols: ▪, 293K; •, 303K; ▴, 313K; ▾, 323K.

The *k_2_*, 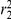 and *q_e_* values were calculated by a plot of *t*/*q_t_* vs *t* and were given in Table 1. The linear plots of *t*/*q_t_* vs *t* were shown in Figure 2 for the pseudo-second order model for adsorption of lactic acid by IRA-67 at 293–323K.

The results in Table 1 revealed that the pseudo-second order kinetic model provided a better correlation for adsorption of lactic acid by IRA-67 compared to the pseudo-first order model, since the 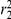 values (0.994–0.999) were much higher than the 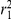 (0.900–0.954) and the experimental *q_e_,_exp_* values were much closer to the theoretical *q_e2_,_cal_* values than to the theoretical *q_e1_,_cal_* values.

### Adsorption isotherm models

Adsorption equilibrium is achieved when the solution concentration does not vary with the contact time. These equilibrium data can be applied to predict the adsorption models and related theories linked to the adsorption equilibrium. In the present study, the Langmuir, Freundlich and Dubinin-Radushkevich (D-R) isotherm models are employed and their expressions are given by Equations (9), (10) and (11), respectively [13], [14], [15].

The Langmuir theory assumes that adsorption takes place at specific homogeneous adsorbent sites and once a lactic acid molecule occupies a site, no further adsorption can occur at the same site. The linear form is given by:
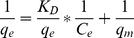
(9)where *C_e_* is the lactic acid concentration at equilibrium (g/liter), *q_m_* is the maximum adsorption capacity of IRA-67 (g lactic acid/g wet resin), *K_D_* is equilibrium constant of the Langmuir isotherm model (g/liter). *K_D_* and *q_m_* were calculated to be 1.148 and 0.1923 (R^2^  =  0.950).

**Figure 3:**
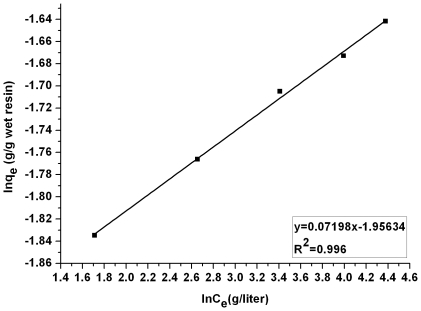
The Freundlich isotherm linear plot for adsorption of lactic acid. Lactic acid concentrations: 21.5, 31.2, 48.4, 73.0, 99.0 g/liter, contact time 24 h, temperature 323K.

The Freundlich adsorption isotherm model assumes that the adsorbent is composed of a heterogeneous adsorption surface with different classes of adsorption sites and the linear form is expressed by:
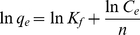
(10)where *K_f_* is a constant related to the adsorption capacity and n is an empirical parameter linked to the adsorption intensity varying with the heterogeneity of the adsorbent. The values of *K_f_* and n were 0.14 and 13.89 according to the plot of ln*q_e_* vs ln*C_e_* presented in Figure 3 (R^2^  =  0.996).

The D-R isotherm model is more frequently used to determine whether the nature of adsorption process is physical or chemical, when the equilibrium data are submitted. The linear equation of the D-R isotherm model is represented as:
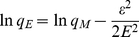
(11)where *q_E_* is the equilibrium concentration of lactic acid adsorbed on IRA-67 (mol/g), *q_M_* is the maximum lactic acid adsorption capacity (mol/g), *E* is the adsorption mean free energy (J/mol) and ε represents the Polanyi potential (*ε  =  RT*ln(*1 + 1/C_e_*)). *q_M_* and *E* were calculated to be 2.140*10^−3^ mol/g and 12.18 kJ/mol (R^2^  =  0.940) by the ln*q_E_* vs *ε^2^* plot.

The results from the D-R isotherm model revealed that the adsorption of lactic acid was a chemical reaction process, since the obtained value of adsorption mean free energy *E* lay within 8–16 kJ/mol in this case [16]. Although both the Langmuir and D-R isotherm models were to some extent able to describe the relationship between the amount of equilibrium adsorption and equilibrium concentration of lactic acid, via contrasting the value of correlation coefficient R^2^, the Freundlich adsorption isotherm model was found to perfectly simulate the relationship between *q_e_* and *C_e_* and the n value (n≫1) indicated that the adsorption of lactic acid by IRA-67 was favorable under the studied condition.

### Effects of resin dose and lactic acid concentration

**Figure 4:**
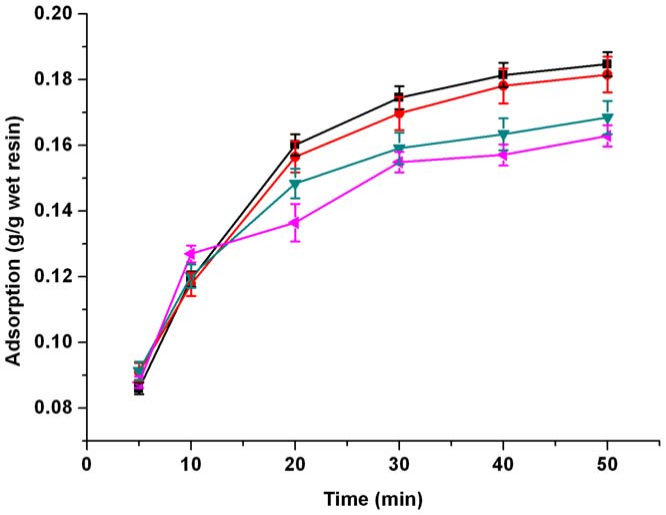
Effect of resin dose on adsorption of lactic acid. Lactic acid concentration 42.5 g/liter, temperature 323K. Symbols: ▪, 0.50 g; •, 0.75 g; ▾, 1.50 g; ◂, 1.75 g.

**Figure 5:**
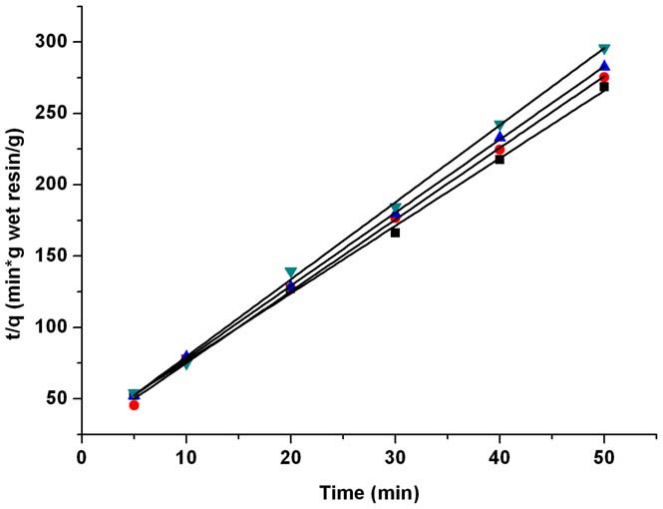
Pseudo-second order kinetics for adsorption of lactic acid at various resin doses. Lactic acid concentration 42.5 g/liter, temperature 323K. Symbols: ▪, 0.50 g; •, 0.75 g; ▴, 1.50 g; ▾, 1.75 g.

The effects of IRA-67 dose and lactic acid concentration were studied. Figures 4 and 5 showed a series of contact time curves with wet resin dose varying from 0.5 to 1.75 g/10 ml. Similar plots were also obtained with lactic acid concentrations from 21.5 to 99.0 g/liter. Table 2 exhibited the results as a plot of *t/q_t_* against *t* for adsorption of lactic acid for the pseudo-second order model with the correlation parameters of pseudo-second order rate, as well as the adsorption rate constant *k_2_*, initial adsorption rate *h*, and the equilibrium adsorption capacity *q_2_* as functions of resin dose and lactic acid concentration. Similar results of pseudo-first order model were also obtained (Table S1). *k_2_* and *h* for IRA-67 showed an increase but *q_2_* decreased with an increase in the above resin dose. On the other hand, *k_2_*, *h* and *q_2_* showed an increase with the lactic acid loading concentrations from 21.5 to 99.0 g/liter.

**Table 2 pone-0013948-t002:** Kinetic parameters of pseudo-second order reaction at various resin doses and lactic acid concentrations.

Lactic acid g/liter	*m_s_* g/10 ml	Temperature K	*q_2_* g/g_wr_	*k_2_*	*h* g/(g_wr_·min)	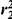
42.5	0.50	323	0.2138	0.6401	0.02927	0.999
42.5	0.75	323	0.2010	0.8086	0.03268	0.998
42.5	1.50	323	0.1859	1.0289	0.03555	0.998
42.5	1.75	323	0.1762	1.2003	0.03725	0.998
21.5	1.00	323	0.1947	0.4884	0.01852	0.996
48.4	1.00	323	0.2056	0.6295	0.02661	0.997
73.0	1.00	323	0.2088	0.8192	0.03572	0.999
99.0	1.00	323	0.2126	0.9483	0.04288	0.999

The corresponding linear curves of the values of *q_2_* and *h* can be respectively depicted as a function of *m_s_* or *C_0_* for IRA-67 by [12]:
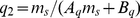
(12)

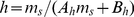
(13)

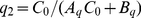
(14)

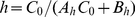
(15)substituting the values of *q_2_* and *h* from Table 2 respectively into Equations (12), (13), (14) and (15) to obtain the empirical parameters listed in Table 3 and then into Equations (7) and (8), respectively. Finally, the relationships of *q_t_* against *m_s_*, *t* and *q_t_* against *C_0_*, *t* are expressed as follows:
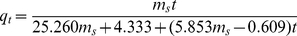
(16)

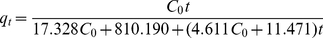
(17)


**Table 3:** Empirical parameters for predicted *q_2_* and *h* from *m_s_* and *C_0_*.

	*A_q_* g/g_wr_	*B_q_* g/10 ml	*R^2^*	*A_h_* g_wr_/(g·min)	*B_h_* g_wr_^2^/(g•10 ml)	*R^2^*
**Resin dose**	5.853	−0.609	0.942	25.260	4.333	0.982
**Lactic acid concentration**	4.611	11.47	0.985	17.33	810.19	0.948

**Figure 6:**
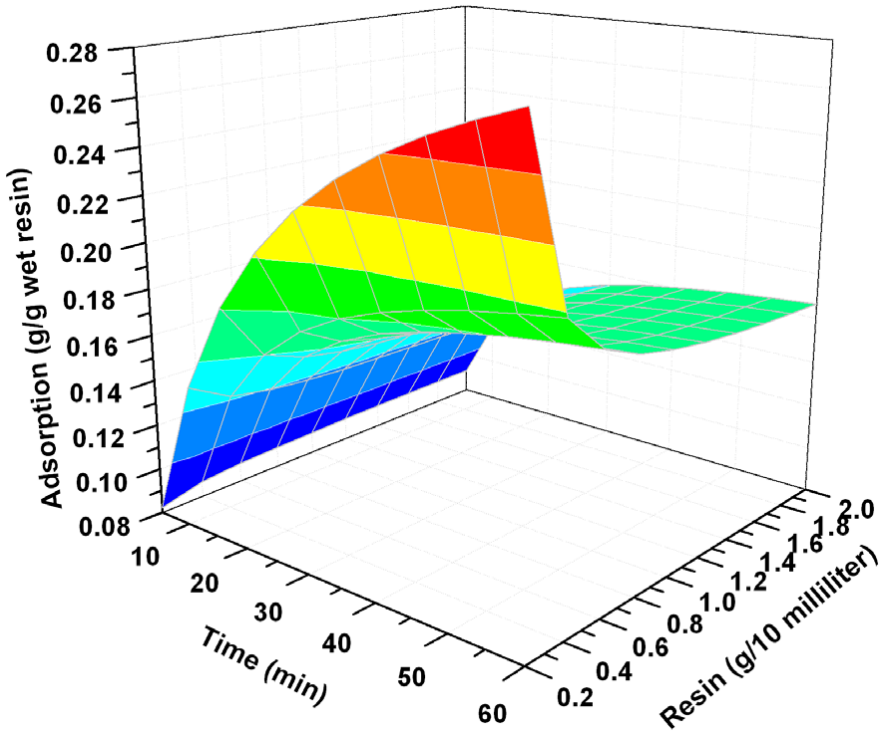
Effect of resin dose on adsorption of lactic acid onto resin at different contact times. Lactic acid concentration 42.5 g/liter, temperature 323K.

**Figure 7:**
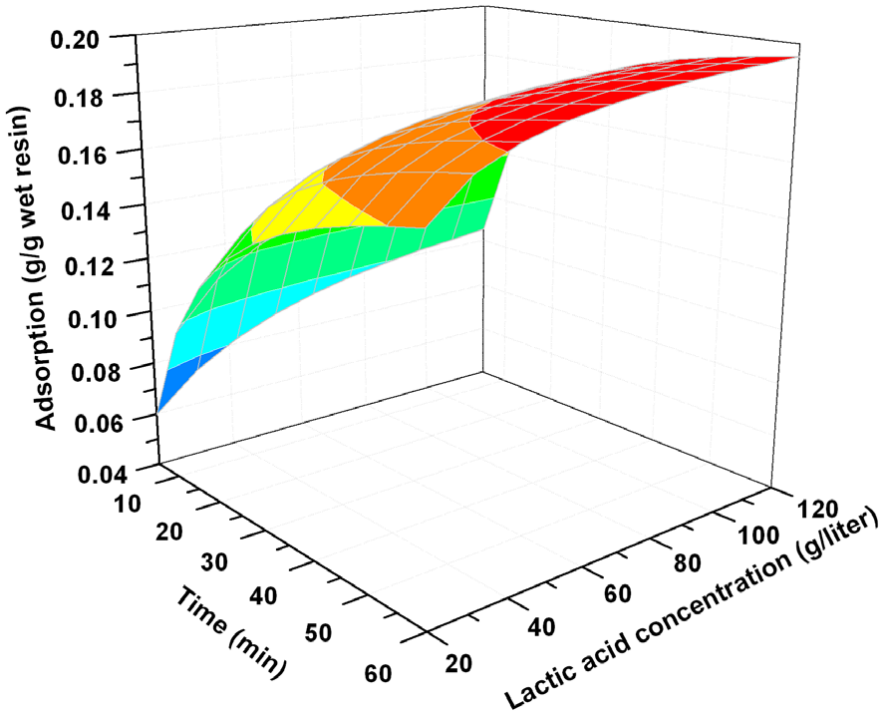
Effect of lactic acid concentration on its adsorption onto resin at different contact times. Resin dose 1 g/10 ml, temperature 323K.

These equations can be used to predict the amount of lactic acid adsorption at any reaction time at the given resin dose and lactic acid concentration. The three dimensional plots of Equations (16) and (17) were shown in Figures 6 and 7.

### Adsorption mechanisms

In the adsorption process, either the film diffusion or the intraparticle diffusion will control the overall rate of adsorption. It is very useful to predict the rate-limiting step of the adsorption process by studying the resin application. The dynamic data have been analyzed by the model given by Boyd [17] as expressed by the following equations:
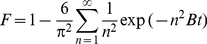
(18)

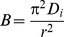
(19)

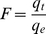
(20)where *F* is the fractional attainment of equilibrium at time *t* (min), *D_i_* (cm^2^/s) is the effective diffusion coefficient of lactic acid in the resin phase, and *r* (cm) is the radius of the resin particle.

The values of *F* can be calculated using Equation (20). A value of *Bt* corresponds with each value of *F* by Equation (18) and the values of *D_i_* can be obtained by Equation (19). The control mechanism of either film diffusion or intraparticle diffusion for lactic acid adsorption by IRA-67 can be determined by the linear plots of *Bt* vs *t*. The results in Table 4 demonstrated that at the lower lactic acid concentrations (<50 g/liter), the adsorption was an intraparticle diffusion, since the linear plots passed through the origin. However, at the higher concentrations (>70 g/liter), the linear plots did not pass through the origin, which indicated that the adsorption was a film diffusion. Moreover, the value of *F* had nothing with the lactic acid concentration and was only dependent on the *D_i_/r^2^* ratio. In addition, the values of *D_i_* changed very little with the increase of lactic acid concentration but increased with the rise of temperature. The reason is that the increased mobility of the ingoing resin at the higher temperature surpassed the effluence of retarding forces [18].

**Table 4:** Parameters of Boyd model at various lactic acid concentrations and temperatures.

323K				42.5 g/liter			
Lactic acid g/liter	*B* /min	*D_i_* cm^2^/s	*R^2^*	Temperature K	*B* /min	*D_i_* cm^2^/s	*R^2^*
21.5	0.042	8.67E-08	0.921	293	0.026	5.41E-08	0.980
48.4	0.046	9.54E-08	0.995	303	0.031	6.31E-08	0.910
73.0	0.045	9.30E-08	0.992	313	0.036	7.54E-08	0.986
99.0	0.047	9.67E-08	0.964	323	0.046	9.42E-08	0.982

### Adsorption thermodynamics

**Figure 8:**
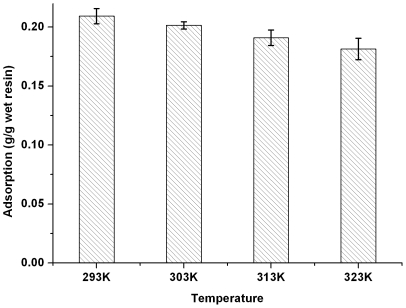
Effect of temperature on adsorptive capacity of IRA-67. Resin dose 1 g/10 ml, lactic acid concentration 42.5 g/liter.

Figure 8 showed the change of adsorption at different temperatures. According to the chart, the amount of adsorption decreased with the increase of temperature, but the variation was not large. In order to study the thermodynamic behavior of lactic acid adsorption by IRA-67, thermodynamic parameters such as changes in free energy (*ΔG°*), enthalpy (*ΔH°*) and entropy (*ΔS°*) were applied. For the reaction *R↔R_LA_*, based on the theory described by Beard and Qian [19], we deduced for the first time the formula for calculating the change of Gibbs free energy (*ΔG*) as follows:
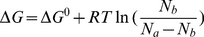
(21)where *N_a_* is the total number of effective binding sites at different temperature and lactic acid loading concentration, and *N_b_* is the number of effective binding sites occupied by lactic acid at different contact times. In this equation, the values of *N_a_* and *N_b_* are affected by both the operating temperature and lactic acid concentration. However, in the following calculations, it is found that the value of *N_b_/*(*N_a_−N_b_*) is only linked to temperature. When the reaction reaches the equilibrium state at *ΔG*  =  0, then *ΔG°*, *ΔH°* and *ΔS°* are calculated by the following equations:
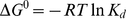
(22)

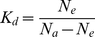
(23)

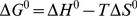
(24)

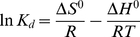
(25)where *K_d_* is the distribution coefficient for the adsorption, *N_e_* is the number of effective binding sites occupied by lactic acid in equilibrium state, *T* is the solution temperature (*K*) and *R* is universal gas constant of 8.314 J/(mol•K).

The distribution coefficient *K_d_* could be also obtained by plotting ln(*C_e_/q_e_*) vs *C_e_* and elongating to zero *C_e_* as suggested by Khan and Singh [20]. However, in the following calculation with such *K_d_* values, the R^2^ value of ln*K_d_* vs *1/T* plot is lower than when using Equation (21). Perhaps the main reason is that it is very hard to obtain a high value of R^2^ from plots of ln(*C_e_/q_e_*) vs *C_e_*. Since this indicates the capability of the resin to retain lactic acid, *K_d_* would be a useful parameter to compare the adsorptive capacities of different resins for lactic acid, if measured under same experimental conditions [21].

**Figure 9:**
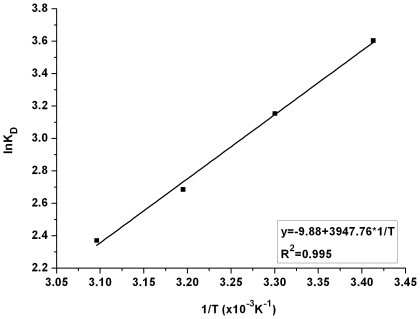
Plot of ln*K_d_* versus *1/T* at lactic acid concentration of 42.5 g/liter.

*ΔG°* can be determined by Equation (22), *ΔH°* and *ΔS°* were respectively calculated from the slope and intercept of van't Hoff plots of ln*K_d_* vs *1/T* as presented in Figure 9. The change in Gibbs free energy (*ΔG°*) for adsorption of lactic acid onto IRA-67 was available with an initial concentration of 42.5 g/liter lactic acid and the results were given in Table 5. The obtained negative values of *ΔG°* indicated that the adsorption of lactic acid onto IRA-67 was spontaneous. Furthermore, the negative *ΔG°* descending with the increase of temperature indicated a decrease in adsorption at higher temperatures [22]. The negative value of enthalpy *ΔH°*, calculated to be −32.82 kJ/mol, indicated that the adsorption processes was exothermic in nature at 293–323K. The obtained negative value of *ΔS°* exhibited the decreased randomness at the solid-solution interface during the adsorption of lactic acid on the active sites of IRA-67. It was also noted that, although decreasing against the rising temperature, the values of *K_d_* were still large at a relatively high temperature, which supported the possibility of industrial lactic acid adsorption by IRA-67.

**Table 5:** Thermodynamic parameters for adsorption of lactic acid onto Amberlite IRA-67 at different temperatures.

Temperature K	*ΔG°* kJ/mol	*K_d_*	*ΔS°* kJ/(mol•K)	*ΔH°* kJ/mol
293	−8.78	36.72	−0.82	−32.82
303	−7.94	23.42	−0.82	−32.82
313	−6.99	14.67	−0.82	−32.82
323	−6.73	10.71	−0.82	−32.82

## Discussion

We have described the dynamic equilibrium and thermodynamic evaluation on adsorption of lactic acid by the weakly basic resin IRA-67. The pseudo-second order model fits well with the kinetic data, in which the equilibrium and ultimate adsorption are 0.182 g and 0.199 g at 323K, respectively. Moreover, the Freundlich isotherm model fits the equilibrium data well, which indicates that the adsorption process is heterogeneous. Therefore, the lactic acid molecule is absorbed first on the higher energy site prior to the lower energy site. The resin dose and lactic acid loading concentration respectively somehow exhibit negative and positive effects on unit resin adsorption, and it is possible to predict the adsorption amount of lactic acid at any time with various resin doses and lactic acid loading concentrations.

It is first found that the overall rate of adsorption is mainly controlled by the intraparticle diffusion at a lower lactic acid concentration (<50 g/liter) but is controlled by the film diffusion at a relatively higher concentration (>70 g/liter) via a slightly increased *D_i_* with the increase of temperature. Thus, the effect of temperature on lactic acid adsorption is very limited. In this research, Equation (21) could be favorably matched the formula in reference [20] and provides an easier alternative way to determine resin adsorption thermodynamics. The adsorption process for lactic acid is spontaneous and exothermic. Also, the values of the distribution coefficient *K_d_* changes inversely with temperature resulting in a slightly decreased amount of lactic acid adsorbed on the resin. However, the overall value is still large at a relatively high temperature due to the fact that the distribution coefficient demonstrates the capability of the resin to retain lactic acid, and the higher the value of *D_i_*, the lower is the adsorption of lactic acid.

To date, the simultaneous removal of lactic acid in the fermentation has not been carried out as an industrial application. The fatal limitation is that during the *in situ* product removal process of lactic acid, it is fairly difficult to avoid the risk of contamination caused by other bacteria. The reason is that the optimal fermentation temperature for most industrial strains is in the range of 303K to 315K. To achieve continued lactic acid recovery on the industrial scale, one of the most likely ways is to use thermophilic producers and to efficiently adsorb the lactic acid by resin at high temperature, thus dramatically reducing the possibility of contamination. Finally, the results of this work indicate that, due to the characteristics of quick and high adsorption especially at a high temperature, use of IRA-67 is possible for *in situ* product removal of lactic acid. This, coupled with the use of thermophilic producer, would allow low-carbon clean manufacture without contamination by other bacteria. Thus, the production cost would sharply decrease and environmental pollution would be avoided.

## Materials and Methods

### Materials

Amberlite IRA-67 (Rohm and Hass Company, USA), a weakly basic gel-type polyacrylic resin with a functional tertiary amine group, was selected for this study. L-lactic acid (purity>98%, Sigma-Aldrich Corporation, USA) was used as HPLC standard and a conventional lactic acid reagent of AR grade (purity  =  85–90%, Tianjin Guangfu Fine Chemical Institute, China) was used for sorption experiments.

### Adsorption kinetics and equilibrium studies

Due to the resin's nature, Amberlite IRA-67 hardly occurs in the OH^−^ form. Before utilization, the resin was treated with 4% NaOH for 24 h, then washed with double-distilled water. One gram of wet Amberlite IRA-67 was mixed with 10 ml of 42.5 g/liter lactic acid solution and shaken at 150 rpm in a 100-ml conical flask. Duplicate samples were taken at various time intervals at 293, 303, 313 and 323K. The equilibrium was achieved at 24 h. The amount of lactic acid adsorbed onto the resin at equilibrium was calculated from the mass balance of the equation as given below:
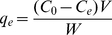
(26)where *C_0_* and *C_e_* are the initial and equilibrium concentrations of lactic acid solution (g/liter), *q_e_* is the amount of lactic acid adsorbed at equilibrium (g/g wet resin), *V* is the volume of the lactic acid solution (liter), and *W* is the mass of the resin used (g).

### Desorption process

Different characteristics of resins require different elution protocols for desorption. For IRA-67 resin (free base form), NaOH (2–4%), NH_4_OH (2–4%) and Na_2_CO_3_ (4–8%) are the commonly used regenerants. In this study, 4% NaOH was used to completely desorb the adsorbed lactic acid with a contact time of 24 h.

### Analytical methods

Lactic acid concentration was determined using an Agilent 1100 series high-performance liquid chromatography (HPLC) system (Hewlett-Packard Corporation, USA), a VWD UV detector, and a Bio-Rad Aminex HPX-87H column maintained with a thermostat at 323K. The mobile phase was 5 mM sulfuric acid at a flow rate of 0.60 ml/min.

## Supporting Information

Table S1 Kinetic parameters of pseudo-first-order reaction at various resin doses and lactic acid concentrations.
(0.03 MB DOC)

## References

[1] El-Beyrouty C, Huang V, Darnold CJ, Clay PG (2006). Poly-L-lactic acid for facial lipoatrophy in HIV.. Ann Pharmacother.

[2] Kasuga T, Ota Y, Nogami M, Abe Y (2001). Preparation and mechanical properties of poly lactic acid composites containing hydroxyapatite fibers.. Biomaterials.

[3] Qin J, Zhao B, Wang X, Wang L, Yu B (2009). Non-sterilized fermentative production of polymer-grade L-lactic acid by a newly isolated thermophilic strain *Bacillus* sp. 2–6.. PLoS ONE.

[4] Wang E, Hatanaka H, Iijima S, Tokebayashi T, Shi Z (1988). Control of cell and lactate concentration in a hollow fiber bioreactor for lactic acid fermentation.. J Chem Eng Jpn.

[5] Chen CC, Ju LK (1998). Adsorption characteristics of polyvinylpyridine and activated carbon for lactic acid recovery from fermentation of *Lactobacillus delbrueckii*.. Sep Sci Technol.

[6] Chabani M, Amrane A, Bensmaili A (2009). Equilibrium sorption isotherms for nitrate on resin Amberlite IRA 400.. J Hazard Mater.

[7] Srivastava A, Roychoudhury K, Sahai V (1992). Extractive lactic acid fermentation using ion exchange resin.. Biotechnol Bioeng.

[8] Moldes AB, Alonso JL, Parajo JC (2003). Recovery of lactic acid from simultaneous saccharification and fermentation media using anion exchange resins.. Bioproc Biosyst Eng.

[9] Uslu H, İnici İ, Bayazit ŞS (2010). Adsorption equilibrium data for acetic acid and glycolic acid onto Amberlite IRA-67.. J Chem Eng Data.

[10] Patel M, Bassi A, Zhu JJX, Gomaa H (2008). Investigation of a dual-particle liquid-solid circulating fluidized bed bioreactor for extractive fermentation of lactic acid.. Biotechnol Progr.

[11] Ho YS, McKay G (1999). Pseudo-second order model for sorption process.. Process Biochem.

[12] Ho YS, McKay G (1999). A kinetic study of dye sorption by biosorbent waste product pith.. Resour Conserv Recy.

[13] Langmuir I (1918). The adsorption of gases on plane surfaces of glass, mica and platinum.. J Am Chem Soc.

[14] Freundlich H (1906). Adsorption in solution.. Z Phys Chem.

[15] Dubinin MM, Zaverina ED, Radushkevich LV (1947). Sorption and structure of active carbons. I. Adsorption of organic vapors.. Zhurnal Fizicheskoi Khimii.

[16] Helfferich F (1962). Ion Exchange.

[17] Boyd GE, Adamson AW, Myers LS (1947). The exchange adsorption of ions from aqueous solutions by organic zeolites. II. Kinetics.. J Am Chem Soc.

[18] Gupta VK, Ali I (2001). Removal of DDD and DDE from wastewater using bagasse fly ash, a sugar industry waste.. Wat Res.

[19] Beard DA, Qian H (2007). Relationship between thermodynamic driving force and one-way fluxes in reversible processes.. PLoS ONE.

[20] Khan AA, Singh RP (1987). Adsorption thermodynamics of carbofuran on Sn (IV) arsenosilicate in H^+^, Na^+^, and Ca^2+^ forms.. Colloids Surf.

[21] Fontes MPF, Gomes PC (2003). Simultaneous competitive adsorption of heavy metals by the mineral matrix of tropical soils.. Appl Geochem.

[22] Sarl A, Tuzen M (2009). Equilibrium, thermodynamic and kinetic studies on aluminum biosorption from aqueous solution by brown algae (*Padina pavonica*) biomass.. J Hazard Mater.

