# Supplementary material for: The Role of Lactic Acid Adsorption by Ion Exchange Chromatography
Source: PLoS One. 2010 Nov 11;5(11):e13948. doi: 10.1371/journal.pone.0013948 (PMC2978715; doi:10.1371/journal.pone.0013948)
Supplement: Table S1 — Kinetic parameters of pseudo-first-order reaction at various resin doses and lactic acid concentrations. (0.03 MB DOC) [file pone.0013948.s001.doc]

**Table S1.** Kinetic parameters of pseudo-first-order reaction at various resin doses and lactic acid concentrations.

| **Lactic acid g/liter** | ***m_s_* g/10 ml** | **Temperature K** | ***q_e,exp_* g/g_wr_** | ***q_1_* g/g_wr_** | ***K_1_*** | $r_{1}^{2}$ |
| --- | --- | --- | --- | --- | --- | --- |
| 42.5 | 0.50 | 323 | 0.200972 | 0.12011 | -0.04411 | 0.938 |
| 42.5 | 0.75 | 323 | 0.18894 | 0.10613 | -0.04927 | 0.929 |
| 42.5 | 1.50 | 323 | 0.174746 | 0.09467 | -0.05552 | 0.978 |
| 42.5 | 1.75 | 323 | 0.165628 | 0.09782 | -0.06871 | 0.959 |
| 21.5 | 1.00 | 323 | 0.183018 | 0.12907 | -0.03726 | 0.960 |
| 48.4 | 1.00 | 323 | 0.193264 | 0.12771 | -0.04592 | 0.983 |
| 73.0 | 1.00 | 323 | 0.196272 | 0.11160 | -0.05206 | 0.980 |
| 99.0 | 1.00 | 323 | 0.199844 | 0.10659 | -0.05805 | 0.981 |
